# Drosophila yakuba – Tsc1

**DOI:** 10.17912/micropub.biology.000474

**Published:** 2021-11-12

**Authors:** Bailey Lose, Abigail Myers, Savanah Fondse, Ian Alberts, Joyce Stamm, James J. Youngblom, Chinmay P. Rele, Laura K. Reed

**Affiliations:** 1 The University of Alabama, Tuscaloosa, AL USA; 2 California State University Stanislaus, Turlock, CA USA; 3 University of Evansville, Evansville, IN USA

## Abstract

Gene Model for the ortholog of *Tsc1* in the *Drosophila yakuba* DyakCAF1 assembly (GCA_000005975.1).

**Figure 1:**
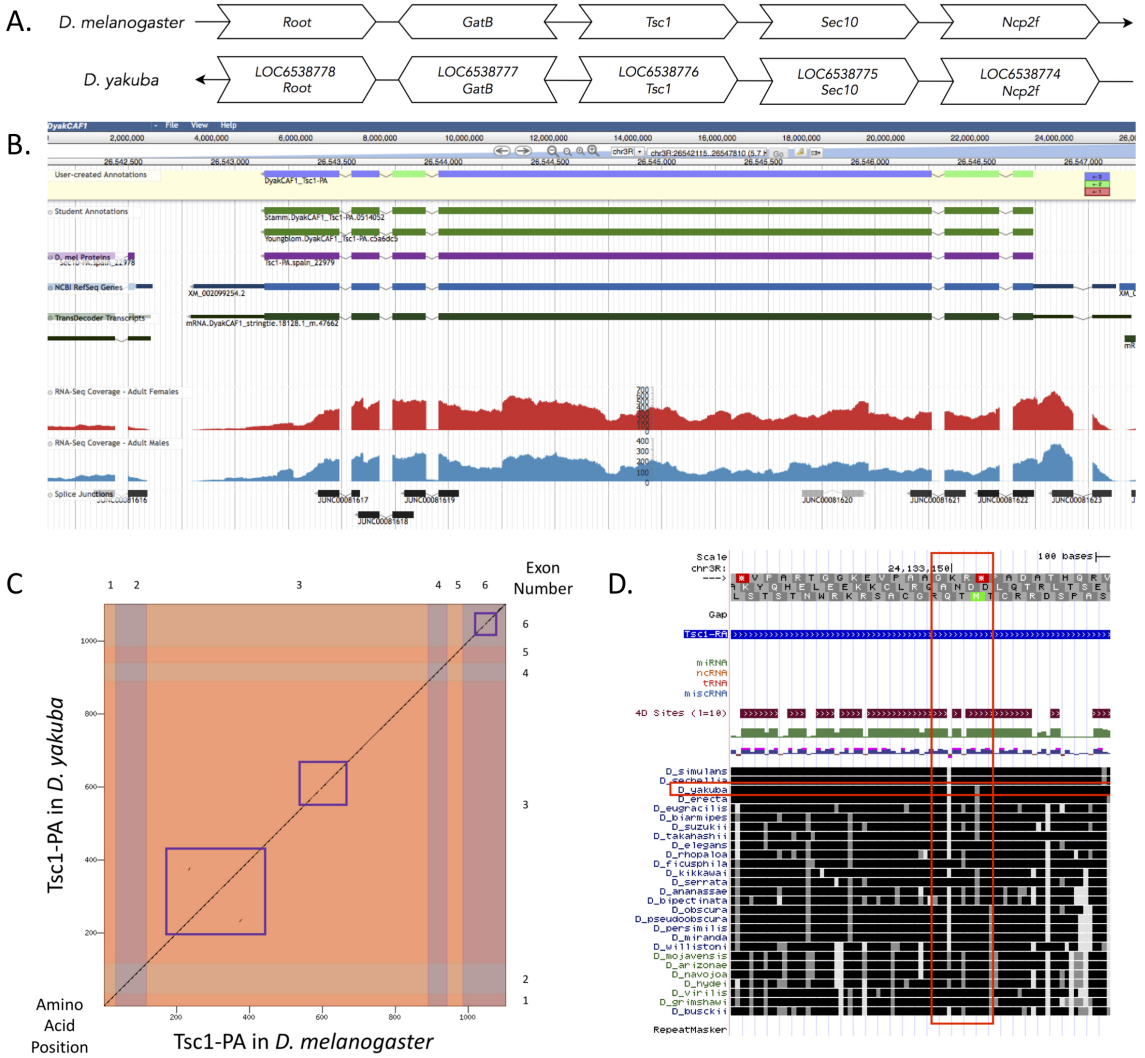
(A) Synteny between *D. melanogaster* and *D. yakuba* in the genomic neighborhood around our focal gene, *Tsc1*: the thin arrows at the back indicate the strand in each species, whereas the thick arrows with the gene names in them indicate direction relative to *Tsc1*. The top line of text in the *D. yakuba* gene arrows indicates the locus identifiers specific to *D. yakuba* genes while the bottom line of text indicates the orthologous gene in *D. melanogaster*; (B) Gene Model in Apollo: A screenshot of the Apollo instance housing the gene model, containing student annotations, D. mel Proteins, NCBI RefSeq Genes, TransDecoder Transcripts, RNA-Seq tracks (Yang *et al.*, 2018; SRP006203) and splice junctions, exon reading frames are indicated in blue, green, and red as in legend; (C) Dot Plot of gene in *D. melanogaster* (*x*-axis) vs. the gene in *D. yakuba* (*y*-axis), the numbers on the bottom and left correspond to amino acid position, and the numbers on the top and right correspond to exon number, the vertical and horizontal stripes of color highlight the exon corresponding to each number, the purple boxes represent a lack of sequence similarity in the protein sequences within coding exons three and six; (D) An image of exon three in the gene model from the GEP mirror of the UCSC Genome Browser for *D. yakuba*. The Conservation Track of 28 *Drosophila* species compared to exon three in *D. melanogaster* *Tsc1-RA* contains regions lacking sequence similarity (vertical red box; *D. yakuba* is highlighted in the horizontal red box). The gray scale at the top of the image represents the three reading frames, where *Tsc1-RA* is in reading frame +2 of *Drosophila melanogaster*. In the grayscale, the red boxes are stop codons and the green represent start codons. The maroon, green, and purple/pink tracks above the species alignments represent the ROAST alignments and conservation (28 *Drosophila* species), PhastCons Scores Based on Four-fold Degenerate Sites, and PhyloP Scores Based on Four-fold Degenerate Sites, respectively. For the *Drosophila* conservation track for 28 *Drosophila* species at the bottom of the figure, darker values to indicate higher levels of overall conservation as scored by phastCons.

## Description

Introduction

*Tsc1* (*LOC6538776*)in *D. yakuba* is an ortholog to the *Tsc1* gene in *D. melanogaster*. We used the *D. yakuba* CAF1 assembly (GCA_000005975.1, Drosophila 12 Genomes Consortium **et al.*,* 2007) and the *D. melanogaster* dm6 assembly (GCA_000001215.4, Release 6.32 FB2021_01). Mutations in either the *Tsc1* or *Tsc2* gene can cause the hamartoma syndrome tuberous sclerosis complex (TSC) (Dabora *et. al*, 2008). These two genes operate together in the insulin signaling pathway as tumor suppressors because of their ability to control cell growth (Gao, 1970). A mutation in the *Tsc1* gene can also cause benign tumors to form in multiple organs (Potter, Huang, Xu, 2001). The NCBI RefSeq predicted model in *D. yakuba,* with a RefSeq accession number of XM_002099254.2 (RefSeq Release 204),has the same number of exons as the *Tsc1* gene (*LOC6538776)* in *D. melanogaster* indicating they have an orthologous relationship. The methods and dataset versions used to establish the gene model are described in Rele *et al.* (2021). The GEP maintains a mirror of the UCSC Genome Browser (Kent WJ *et al.*, 2002; Gonzalez *et al.*, 2020), which is available at https://gander.wustl.edu and contains additional information about data sources and versions.

Synteny

The *Tsc1* gene, located on chromosome 3R in *D. melanogaster*, is neighboring the genes *Root*, *GatB*, *Sec10*, and *Ncp2f.* The best candidate for the *Tsc1* ortholog gene in *D.* *yakuba* based on the *tblastn* search is found on chromosome 3R. The candidate is also surrounded by the genes *LOC6538778, LOC6538777, LOC6538775,* and *LOC6538774* (which are likely orthologous to *Root*, *GatB*, *Sec10*, and *Ncp2f* in *D. melanogaster* respectively, Figure 1A). We performed a *blastp* search of protein sequence XP_002099290.1 in *D. yakuba* against the protein sequences found in the refseq_protein database for *D. melanogaster* and it showed a high percent identity to *Tsc1* in comparison to the second-best hit. After confirming that the genes surrounding *Tsc1* are orthologous between the two species and the *blastp* results indicated a high percent identity for the *Tsc1* gene between the two species, we determined that this region contains the ortholog for *Tsc1* in *D. yakuba.*

Gene Model

*Tsc1* has one isoform in *D. yakuba*, Tsc1-PA, with six exons. There are also six exons in the *Tsc1* gene located in *D. melanogaster.* A *blastp* search of the protein sequence of *Tsc1* in *D. yakuba* against *D. melanogaster* yields only one significant match with a 97.00% identity with only 33 amino acids differing out of 770. There was a small lack of sequence similarity between the protein sequences of the two species in coding exons three and six as is displayed by the purple boxes in the dot plot (Figure 1C). The large lack of sequence similarity in exon six, shown by the red vertical box in Figure 1D, can also be seen in the conservation tracks of 28 different *Drosophila* species in the UCSC Genome Browser. The lack of sequence similarity in exon six is consistent with the lack of a functionally-characterized protein domain in that region of the gene (FB2021_04, released August 17, 2021). The coordinates of the curated gene models can be found in NCBI at GenBank/BankIt using the accession BK014573. These data are also available in Extended Data files below, which are archived in CaltechData.
